# Telomere uncapping by the G-quadruplex ligand RHPS4 inhibits clonogenic tumour cell growth *in vitro* and *in vivo* consistent with a cancer stem cell targeting mechanism

**DOI:** 10.1038/sj.bjc.6603691

**Published:** 2007-04-03

**Authors:** P Phatak, J C Cookson, F Dai, V Smith, R B Gartenhaus, M F G Stevens, A M Burger

**Affiliations:** 1 Department of Pharmacology and Experimental Therapeutics, Marlene and Stewart Greenebaum Cancer Center, School of Medicine, University of Maryland, Baltimore, MD, USA; 2 Centre for Biomolecular Sciences, School of Pharmacy, University of Nottingham, Nottingham, UK; 3 Institute for Experimental Oncology, Freiburg, Germany

**Keywords:** telomere, telomerase, stem cells, G-quadruplex, RHPS4, Taxol

## Abstract

The pentacyclic acridinium methosulfate salt RHPS4 induces the 3′single-stranded guanine-rich telomeric overhang to fold into a G-quadruplex structure. Stabilisation of the latter is incompatible with an attachment of telomerase to the telomere and thus G-quadruplex ligands can effectively inhibit both the catalytic and capping functions of telomerase. In this study, we examined mechanisms underlying telomere uncapping by RHPS4 in uterus carcinoma cells (UXF1138L) with short telomeres and compared the susceptibility of bulk and clonogenic cancer cells to the G-quadruplex ligand. We show that treatment of UXF1138L cells with RHPS4 leads to the displacement of the telomerase catalytic subunit (hTERT) from the nucleus, induction of telomere-initiated DNA-damage signalling and chromosome fusions. We further report that RHPS4 is more potent against cancer cells that grow as colonies in soft agar than cells growing as monolayers. Human cord blood and HEK293T embryonic kidney cell colony forming units, however, were more resistant to RHPS4. RHPS4-treated UXF1138L xenografts had a decreased clonogenicity, showed loss of nuclear hTERT expression and an induction of mitotic abnormalities compared with controls. Although single-agent RHPS4 had limited *in vivo* efficacy, a combination of RHPS4 with the mitotic spindle poison Taxol caused tumour remissions and further enhancement of telomere dysfunction.

Protection of chromosome termini from end-to-end fusion, recombination and degradation is achieved by the telomeres (Blackburn, 1991; Blasco, 2004). A current model proposes that telomeres form ‘a cap’ at the end of chromosomes. The structure adopted by the G-rich 3′-end overhang is thought to involve a G-quadruplex (Williamson, 1994; Parkinson *et al*, 2002) and/or loops after invading the double-stranded region of the telomere (Griffith *et al*, 1999). The physical integrity of the telomere ‘cap’ must be intact to allow cell division to proceed (Blackburn, 2000). Regulated uncapping occurs normally in dividing cells with the crucial property that a functional telomere rapidly switches back to a capped state (Smith and Blackburn, 1999; Blackburn, 2001). The ‘uncapping’ signal for growth arrest, which is triggered when telomere-mediated chromosome end-protection becomes insufficient due to reduction in telomere length and/or damage to telomere structure, has been elucidated recently. It activates the double-strand break (DSB)-mediated DNA damage response pathway, because a short, dysfunctional telomere can resemble a double-strand DNA break (Blackburn, 2000; d'Adda di Fagagna *et al*, 2003; IJpma and Greider, 2003).

In normal somatic cells, which have a finite replicative lifespan, telomeres progressively shorten with successive cell divisions due to the inability of DNA polymerase to replicate DNA fully to the chromosomal end (Hayflick and Moorhead, 1961; Makarov *et al*, 1997). Cells with self-renewal capacity such as stem and cancer cells possess a telomere maintenance mechanism, namely the expression of the telomere-elongating enzyme telomerase, conferring their immortality. The activation of telomerase has also been shown as an early, crucial event in the genesis of tumour from normal cells and is considered a hallmark of cancer (Kim *et al*, 1994; Hahn *et al*, 1999; Hanahan and Weinberg, 2000). Recently it has become evident that telomerase stabilises telomeres independently of its elongation role through an additional ‘capping’ function and appears to mediate cell survival in the presence of various cytotoxic stresses (Blasco, 2002; Masutomi *et al*, 2003; Sung *et al*, 2005).

Since most normal cells lack telomerase, and because marked differences exist in telomere length between telomerase-positive adult stem cells or germ cells (average telomere length ∼15 kb) and cancer cells (∼5 kb), inhibiting telomerase activity and/or interfering with the telomere capping function have arisen as attractive targets for cancer treatment (Burger, 1999; Kelland, 2005; Burger, 2007).

An approach that may be capable of both shortening telomeres and directly causing telomere uncapping is the use of G-quadruplex ligands. The sequestering of the telomere in a G-quadruplex structure inhibits the catalytic lengthening activity of telomerase, which requires the 3′ end to be in a non-folded form (Zahler *et al*, 1991). G-quadruplex structures are readily bound and stabilised by small molecule ligands such as RHPS4 (3,11-difluoro-6,8,13-trimethyl-8*H*-quino[4,3,2-*kl*]acridinium methosulfate, a pentacyclic salt, NSC 714187, Figure 1A) (Gowan *et al*, 2001) and other G-quadruplex ligands (Burger *et al*, 2005; Reed *et al*, 2006; Tahara *et al*, 2006). A characteristic of RHPS4 is a low overall growth-inhibitory activity in short-term cytotoxicity assays such as the 48 h sulforhodamine B assay used by the NCI 60 cell line screen (mean IC_50_ 13.18 *μ*M), but potent inhibition of telomerase enzyme activity (IC_50_ 0.33 *μ*M) (Heald *et al*, 2002). RHPS4, however, exerts clear tumour growth inhibitory effects in longer term growth assays *in vitro* in various experimental models (Gowan *et al*, 2001; Leonetti *et al*, 2004; Cookson *et al*, 2005); phenotypic changes are consistent with a G-quadruplex-stabilizing mechanism of action at telomeres, with the consequent inhibition of telomerase. Sensitivity to growth inhibition by RHPS4 appears correlated to telomere length as shown in a panel of human tumour lines that were grown in the clonogenic assay, also known as the human tumour stem cell assay (HTCA) (Hamburger and Salmon, 1977; Cookson *et al*, 2005). The most sensitive tumour line was the uterus carcinoma UXF1138L, which possesses short telomeres (mean TRF 2.7 kb). UXF1138L cells were thus selected for *in vitro* experiments and *in vivo* efficacy testing reported here. We show that treatment of UXF1138L cells with RHPS4 leads to rapid telomere uncapping, DSB DNA-damage signalling and consequently chromosomal end-to-end joining. We further report that RHPS4 is more potent against cancer cells that grow as colonies in soft agar than bulk cancer cells that grow as monolayers. Colonies formed by human cord blood and HEK293T embryonic kidney cells were more resistant to RHPS4. Similarly, *in vivo*-treated UXF1138L xenograft tissue had a decreased clonogenicity and exhibited mitotic abnormalities, consistent with telomere dysfunction. Finally, we demonstrate that the telomere-targeting agent RHPS4 and the tumour ‘debulking’ agent Taxol act in a synergistic manner and can cause complete remission of UXF1138L xenografts.

## MATERIALS AND METHODS

### Drugs

RHPS4 was synthesised as described (Heald *et al*, 2002). RHPS4 is water-soluble and was therefore dissolved in phosphate-buffered saline (PBS). For *in vitro* studies, paclitaxel (Taxol) was purchased from Sigma (St Louis, MO, USA) and dissolved in dimethylsulphoxide; for *in vivo* experiments, the clinical formulation was used and obtained from our Hospital Pharmacy (in Cremophor from Bristol-Myers Squibb, New York, NY, USA).

### Cell lines and animals

The UXF1138L uterus carcinoma cell line was originally established from a patient tumour by Prof Heiner Fiebig at the University of Freiburg, Germany (Fiebig and Burger, 2001). All animal experiments were conducted under an animal license approved by the German Federal Government (Regierungspräsidium Freiburg) and in compliance with the UKCCCR guidelines on experimental neoplasia (Workman *et al*, 1998). Six- to 8-week-old female thymus aplastic nude mice of NMRI genetic background were used for establishment and serial propagation of the human tumour xenograft from the cell line. PC3 and MCF-7 cells were obtained from American Type Culture Collection (Manassas, VA, USA). The HEK293T human embryonic kidney cell line was a kind gift from Dr Arun Seth (Sunnybrook Health Sciences Centre, Toronto, Canada). Umbilical cord blood was freshly obtained from our hospital maternity ward with the consent of the respective patient, specimens were anonymised. The cord blood was collected into a BD Vacutainer CPT™ and the mononuclear fraction isolated by centrifugation following the manufacturers instructions.

### MTT proliferation assay and *in vitro* combination studies

Cells were grown under standard conditions (5% CO_2_/37°C/humidified atmosphere) in their respective recommended media such as RMPI 1640, or DMEM (from Invitrogen, Carlsbad, CA, USA) supplemented with 10% fetal calf serum and passaged routinely. Exponentially growing cells were seeded in 96-well plates (2000 per well) and drugs (RHPS4 or Taxol) were added in concentrations ranging from 0.1 nM to 100 *μ*M the following day. Cell proliferation was determined 5 days after continuous exposure to drug by addition of 3-(4,5-dimethylthiazol-2-yl)-2,5-diphenyltetrazoliumbromide (MTT) (Mosmann, 1983). The conversion of MTT to purple formazan by viable cells was measured using a SynergyHT plate reader (550 nm) and K4C software (BioTEK, Winooski, VT, USA). Growth curves were generated as percent of control and growth inhibitory concentrations 50 and 100% determined.

Drugs were combined at the fixed ratio of their IC_50_ in six concentrations, ranging from 0.01 to 10 *μ*M for RHPS4, and MTT assays were performed as described above. Fractions of affected cells were calculated from the absorbance readouts and entered into the Calcusyn 2.0 software (Biosoft, Ferguson, MO, USA) (Chou and Talalay, 1984); combination index values were extracted.

### Preparation of metaphase spreads

Cells were grown to 70% confluency and treated with 1 *μ*M RHPS4 or PBS (vehicle control) for 24 h in a T75 tissue culture flask. Supernatants were then replaced with media containing 10 *μ*g ml^−1^ Colcemid (Sigma) and incubated for 90–120 min at 37°C. Next, cells were trypsinised and centrifuged at 500 **g** for 5 min; 8 ml of 60 mmol l^−1^ KCl was added to the pellets and the cell suspension incubated for 20 min at 37°C. In a pre-fixation step, 2 ml freshly made fixative (methanol/glacial acid 3 : 1) was added on top of the hypotonic suspension and mixed carefully by turning the tube. After 10 min at RT, the mix was centrifuged at 600 **g** for 10 min and the supernatant removed. For fixation, 10 ml fixative was added and the mix kept at RT for 10 min and centrifuged as above, the step was repeated two more times. Then, 0.5 ml fresh fixative was added to obtain a milky suspension of cells without clumps. Cleaned slides were placed horizontally at a 45° angle and 100 *μ*l of cell solution dropped onto the slide from a distance of about 20 cm. Slides were dried at RT or directly dehydrated through an ethanol series of 70, 90 and 100%. After dehydration, slides were rinsed in PBS and incubated with 0.1 *μ*g ml^−1^ DAPI (4′,6-diamidino-2-phenylindole)/PBS for 30 min at RT. To remove excess DAPI, slides were rinsed × 4 with PBS and mounted (Vectashield, Burlingame, CA, USA). Results were documented using the fluorescent module of a Leica DM4000 microscope with Retiga camera (Leica, Wetzlar, Germany).

### Telomere fluorescence *in situ* hybridisation

All human centromere (cat. no. CP5095-B.5) and telomere (CP5097-DG.5) probes labelled with biotin or digoxigenin (Q-Biogene, Irvine, CA, USA) were used for hybridisation to metaphase preparations of UXF1138L cells following a protocol provided by the manufacturer. The probes were detected with fluorescein-labelled avidin for centromere signal (green), and rhodamine-labelled anti-digoxigenin for telomeres (red/pink). The chromosomes were counterstained with DAPI (blue). Images were captured at × 100 magnification by using a Zeiss Axiovert Fluorescence Microscope (Carl Zeiss, Gottingen, Germany).

### Phosphorylated H2AX (*γ*-H2AX) and hTERT immunofluorescence staining

Approximately 75 000 UXF1138L cells per chamber were seeded onto eight-chamber glass slides (Corning, Acton, MA, USA) 24 h before RHPS4 treatment. After exposure to 1 *μ*M RHPS4 for 1, 6 or 24 h, cells were washed twice with PBS and air-dried. Cells were fixed and permeabilised by immersion into ice-cold methanol/acetone (1 : 1; 3 × 1 min). Slides were blocked overnight at 4°C with 5% bovine serum albumin in PBS and washed with PBS (× 3) before incubation (2 h) with anti-*γ*-H2AX mouse monoclonal antibody (Upstate, Waltham, MA, USA; 1 : 250 in PBS) or hTERT monoclonal antibody (NCL-L-hTERT Novacastra, Newcastle, UK; 1 : 40), respectively. Control cells were probed with mouse IgG (Santa Cruz Biotechnolog Inc., Santa Cruz, CA, USA). Slides were washed 3 × with PBS, before incubation with a goat anti-mouse FITC-conjugated secondary antibody (Sigma; 1 : 100, 3 h). Following further PBS washes (3 ×), slides were incubated with 1 : 5000 DAPI (2 mg ml^−1^; Sigma), washed 3 × in PBS and mounted with Vectashield mounting media. Images were visualised as described above.

### Immunoblotting for *γ*-H2AX

Cells were grown to 50% confluency in six-well plates (BD Falcon, Franklin Lake, NJ, USA) and treated with 1 *μ*M RHPS4 for 1, 6 and 24 h. Histones were released by the method described by Meng *et al* (2005). Briefly, cells were scraped and spun at 2–4°C/1000 **g** for 15 min. Pellets were washed twice with PBS, homogenised with 0.2 N sulphuric acid and centrifuged for 15 min at 2–4°C/13 000 **g**. Supernatants were collected and 0.25 volume of 100% (w/v) trichloroacetic acid was added to precipitate histones. After centrifugation for 15 min at 2–4^o^C/13 000 **g**, pellets were suspended in absolute ethanol for overnight and again spun for 15 min at 2–4°C/13 000 **g**. Histones were dissolved in water and protein concentration was determined using the BioRad protein assay (BioRad Laboratories, Hercules CA, USA). About 12.5 *μ*g of protein were loaded onto 4–20% Tris-glycine gels (Invitrogen) and separated at 125 V for 90 min. Proteins were then transfered onto a polyvinylidene difluoride membrane (Immobilon-P, Millipore, Billerica, MA, USA). Membranes were blocked with 10% non-fat milk in TBS-T (0.02% Tween 20) for 1 h, followed by overnight incubation with *γ*-H2AX (Upstate) antibody (1 : 1000 dilution). Signals were visualised by chemiluminescence using the ECL™ Western-blotting analysis system (Amersham Biosciences, Pittsburgh, PA, USA). Coomassie blue staining was used to assure equal loading control.

### *In vivo* testing

*Single-agent activity*: For the first *in vivo* experiment, tumour fragments (5 × 5 mm) from untreated donor animals were implanted subcutaneously into both flanks of recipient mice. Treatment was initiated 6 days after transplantation (=day 0, median tumour volume of ∼70 mm^3^). Animals were randomised into groups following Lindner's randomisation tables and treated by oral gavage with 5 mg kg^−1^ day RHPS4 or vehicle (PBS), (*n*=5–8 animals per group). In earlier experiments, this dose was found to be the ½ maximal tolerated dose in the mouse strain used and was well tolerated in repetitive dosing regimens. Drug administration was repeated twice weekly for eight times (Q3d × 8) after randomisation. Tumour growth was followed twice weekly by serial caliper measurement, body weights were recorded and tumour volumes were calculated using the standard formula (length × width^2^)/2, where length is the largest dimension and width the smallest dimension perpendicular to the length (Geran *et al*, 1972; Alley *et al*, 2004). The median relative tumour volume was plotted against time. Relative tumour volumes were calculated for each single tumour by dividing the tumour volume on day *X* by that on day 0 (time of randomisation). Growth curves were analysed in terms of tumour inhibition (treated/control, T/C, calculated as median tumour weight of treated divided by median tumour weight of control animals × 100). Statistical data analyses were performed using non-parametrical Wilcoxon Mann–Whitney statistics. Median relative tumour volumes of each treatment group were compared with those of the vehicle control groups. *P*-values <0.05 were considered statistically significant. SPSS 2000, SYSTAT version 10 software, was used.

**Figure 1:**
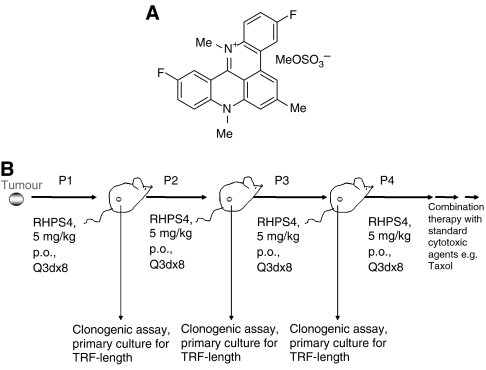
(**A**) Structure of RHPS4 (NSC 714187), 3,11-difluoro-6,8,13-trimethyl-8*H*-quino[4,3,2-*kl*]acridinium methosulfate. (**B**) Design of *in vivo* xenograft studies. Fragments (grey spheres) from an untreated donor animal were implanted into recipient mice, which were treated orally with 5 mg kg^−1^ day^−1^ RHPS4 every 3 days for eight times after randomisation (=6 days after tumour transplantation). Tissue from the vital rim of three tumours from each group was homogenised, digested and primary cultures as well as clonogenic growth assays prepared from single-cell suspensions. Primary cultures were used for analysis of telomere length. The control mouse group was always derived from untreated tumour fragments, but from the same initial passage and donor mouse as were the RHPS4-treated tumours. A total of four passages were analysed.

Upon termination of the experiment, which was when control tumours reached a volume of 1.5 cm in diameter (day 28), RHPS4-treated tumour tissue and control tumours were excised, minced and digested using a mixture of collagenase (123 U ml^−1^), DNase (375 U ml^−1^) and hyaluronidase (290 U ml^−1^) in RPMI 1640 medium at 37°C for 3 h. All enzymes were purchased from Roche (Indianapolis, IN, USA). Primary cultures as well as clonogenic growth assays were prepared from the resulting single-cell suspensions. Primary cultures were used for analysis of telomere length. In addition, RHPS4-treated tumours (5 mg kg^−1^ day^−1^) and control were propagated into new animals for up to three times. The control mouse group was always derived from untreated tumour fragments, but from the same initial passage as were the RHPS4-treated tumours (Figure 1B).

*Combination treatment with Taxol*: After four serial propagations of RHPS4-treated tumour tissue in nude mice (Figure 1), RHPS4 was combined with Taxol. Single-agent Taxol was given at 20 mg^−1^ kg^−1^ i.v. on days 1 and 15. In combination with RHPS4 (given at 5 mg kg^−1^ p.o. twice weekly), only a single dose of Taxol (20 mg kg^−1^ i.v.) was administered on day 1. Tumour growth parameters and body weight were assessed as described above.

Upon termination of the experiments, tumours from three mice per group were excised and immediately fixed in 10% PBS-buffered formalin for 24 h followed by routine paraffin embedding procedures (Burger *et al*, 2005).

### Immunohistochemistry

About 5-*μ*m paraffin sections were cut, dewaxed and antigen retrieval performed in citrate buffer (pH 6.0) in the microwave for 30 min. Sections were then treated with methanol/3% hydrogen peroxide to remove endogenous peroxidase and blocked with 10% normal goat serum in PBS and stained. PBS was used as washing buffer. Cells were incubated overnight at 4^o^C with a monoclonal anti-hTERT antibody (class IgG2a, kappa, Novacastra, Newcastle, UK) diluted 1 : 40 in PBS. Mouse immunoglobulin G2a isotype control (Santa Cruz) was used as negative control. hTERT-specific immunoperoxidase staining was developed using the DAKO Envision+ system (Envision 3,3V-diaminobenzidine Plus kit mouse, DAKO Cytomation). To enhance contrast, tissues were counterstained with haematoxylin. hTERT-specific staining intensity was documented using a Leica DM4000 microscope and digital camera. Sections were viewed and evaluated by two independent pathologists. Mean numbers of atypical mitoses were counted in control and treated tissues from three tumours (four fields of 250 cells per tumour) for the three groups (Burger *et al*, 2005). Box plots were generated using SigmaPlot version 10 software and statistical significance between treatments calculated in SigmaPlot using the Student's *t*-test.

### HTCA/clonogenic assay

Digested tumour tissue from *in vivo* studies discussed above were washed in medium and passed through sieves and the resulting single-cell suspensions seeded into soft agar (*n*=3 tumours per group) as described by us before (Fiebig *et al*, 2004). Single-cell suspensions of cell lines were prepared by trypsinisation from cells growing as monolayers on plastic. Briefly, 5000 (HEK293T cells), 10 000 (PC3, MCF-7 and UXF1138L cells) or 50 000 (UXF1138L tumour tissue) vital cells were added to 0.2 ml Iscove's medium/20% fetal bovine serum/0.05% gentamycin containing 0.4% agar and plated on top of a base layer consisting of 0.2 ml medium with 0.75% agar. The next day, the agar layers were fed with Isocve's medium and cultures incubated at 37°C, 7% CO_2_ for approximately 11 days. Vital colonies were stained with 2-(4-iodophenyl)-3-(4-nitrophenyl)-5-phenyltetrazolium chloride (1 mg ml^−1^) 24 h before evaluation, and colonies >70 *μ*m were counted with an automated image analysis system (Omincon FAS IV, BIOSYS GmbH, Karben, Germany). Drug effect was assessed as growth inhibitory concentrations 50 and 70% (IC_50_ and IC_70_). Methylcellulose was used to grow cord blood stem cells instead of soft agar. The seeding density was 20 000 cells well^−1^. Stem cell growth factor supplemented and optimised methylcellulose (Methocult H4434) was purchased from Stem Cell Technologies (Vancouver, CA, USA). Methylcellulose lacking growth factors was used as negative growth control. Statistical significance between treatment groups was evaluated by using the Student's *t*-test.

### Measurement of telomere restriction fragment length

Genomic DNA was isolated from 3- to 7-day primary cultures established from single cell suspensions of control and treated UXF1138L xenograft tissues using the DNeasy Tissue Kit (Qiagen, Hilden, Germany). Southern blotting was performed with the Telo-TAGGG-telomere length kit from Roche (Penzberg, Germany) and analysed as described before (Burger *et al*, 2005; Cookson *et al*, 2005).

## RESULTS

### Effects of RHPS4 on clonogenic cell growth *in vitro*

**Figure 2:**
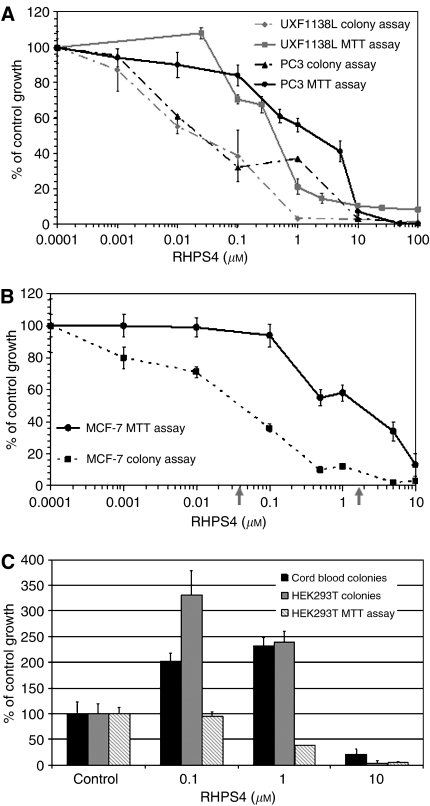
(**A**) Comparison of the antiproliferative activity of RHPS4 in uterus (UXF1138L) and prostate (PC3) cancer cell lines grown as colonies in the HTCA (broken lines) or as monolayers in the MTT assay (solid lines). UXF1138L #of colonies 100%=56; O.D. 100%=0.7±0.03; PC3 #of colonies 100%=94±18, O.D. 100%=0.75±0.01. (**B**) RHPS4 is two log-fold more active in MCF-7 cells grown as colonies (IC_50_=0.04 *μ*M, grey arrow) in the HTCA than in MCF-7 whole-cell populations (IC_50_=2 *μ*M, grey arrow). #of colonies 100%=101±36, O.D. 100%=2.7±0.19. (**C**) Effects of RHPS4 on colonies of HEK293T embryonic kidney cells in the HTCA and human cord blood mononuclear cells in the methylcellulose assay. Colony growth of HEK293T cells is compared to the growth of the bulk cell population by MTT assay. Data are depicted as % of control growth and mean number of colonies per well (HTCA), or the mean optical density measured at 550 nm (MTT assay). All data represent the mean of three independent experiments plus standard deviation. Cord blood #of colonies in the control (100%)=31.6; HEK293T, #of colonies 100%=187.25; O.D. 100%=1.215. Data shown are representative of three independent experiments. O.D.=optical density.

We have compared the growth inhibitory activity of RHPS4 in human bulk tumour cells, by MTT assay, against RHPS4 activity in tumour cells grown as colonies in the clonogenic assay, also termed as HTCA (Figure 2A and B). HEK293T human embryonic kidney cells grown in the HTCA, and cord blood cells cultured in methylcellulose were also treated with RHPS4 (Figure 2C). The MTT assay is a 5-day proliferation test measuring effects on a morphologically heterogeneous, differentiated cell population (bulk cells), whereas the HTCA and methylcellulose assays are longer term (10–15 days) tests in which only a very small fraction of a bulk culture (∼0.1–1%) will grow as colonies. Cells growing anchorage independently as colonies in a semi-solid matrix are considered to be pluripotent stem cells (Hamburger and Salmon, 1977; Fiebig *et al*, 2004; Locke *et al*, 2005). Figure 2A shows a comparison of responses to RHPS4 in two tumour cell lines with short telomeres, the uterus carcinoma UXF1138L and the prostate cancer cell line PC3. Drug concentrations needed to inhibit colony growth in the HTCA were a magnitude of around 20–60-fold lower (IC_50_ UXF1138L=0.02 *μ*M, PC3=0.03 *μ*M) than those needed to cause 50% growth inhibition of the bulk population by MTT assay (IC_50_ UXF1138L=0.4 *μ*M, PC3=1.8 *μ*M). Similar observations were made with the breast cancer cell line MCF-7 (shown in Figure 2B, HTCA IC_50_=0.04 *μ*M; bulk cell IC_50_=2 *μ*M). These data suggest that cancer stem cells are more sensitive to RHPS4 than the whole cancer cell population.

To assess RHPS4 effects on normal stem cells, we exposed the human embryonic kidney cell line HEK293T to drug in the MTT and HTCA assays, and tested RHPS4 effects on colony forming units of the mononuclear cell fraction of human cord blood in methyl cellulose (Figure 2C). The cord blood colony assay was performed with and without colony stimulating growth factors, only methylcellulose containing growth factors grew colonies. Interestingly, RHPS4 concentrations that inhibited colony formation by human embryonic kidney and cord blood (>1 *μ*M) cells were over 25-fold above those inhibiting tumour cell colony formation (Figure 2C). Additionally, in normal cell types as compared with tumour cells, low and pharmacodynamically relevant concentrations of RHPS4 (0.01–1 *μ*M) induced colony formation (Figure 2C). To assure that the induction of colony growth by RHPS4 in normal stem cells is reproducible, we used cord blood from three different individuals and HEK293T cells from different passages. Data shown in Figure 2C represent the mean and standard deviation from three independent experiments. Consistently, 0.1 and 1 *μ*M RHPS4 caused a stimulation of growth by doubling to tripling the number of colonies compared to vehicle (PBS)-treated controls (Figure 2C). However, the plating efficiency (actual number of colonies growing per total number of cells seeded) varied among the experiments and therefore the results are shown as % of control growth. For example, HEK293T control colony growth ranged from a mean number of 54–463 colonies per well, but the least percentage (cut-off level) of growth induction by RHPS4 observed in either the individual HEK293T or the cord blood experiments was 150 %. In contrast, HEK293T cells grown as monolayer cultures in the MTT assay showed no induction of growth at any of the eight dose levels tested (0.001–50 *μ*M). Instead, at RHPS4 levels that induced colony growth 2.4-fold (1 *μ*M), bulk cell growth was inhibited to 60% of control (Figure 2C).

### Effects of RHPS4 on bulk tumour and clonogenic tumuor cell growth *in vivo*

**Figure 3:**
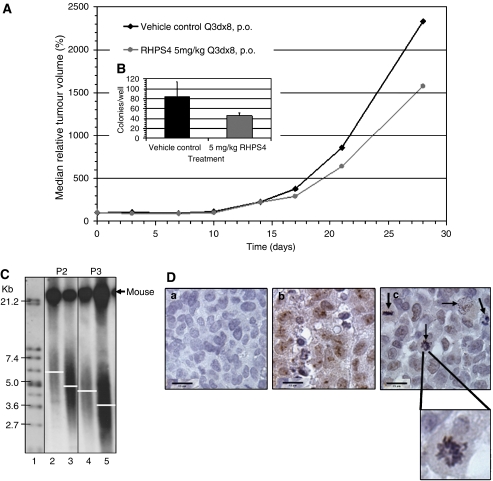
(**A**) Tumour growth inhibition of subcutaneous UXF1138L xenografts in passage 3 of chronic RHPS4 exposure or vehicle only treated controls. Drug was given orally every 3 days, eight times. Shown are the median relative tumour volumes in %; the tumour size at randomisation was set as 100%. (**B**) Effects of RHPS4 *in vivo* treatment on tumour colony growth/stem cell formation *in vitro* from tumours in A. Colony count: control=84±s.d. 29.6; RHPS4 5 mg kg^−1^ day^−1^=46±s.d. 5.1. (**C**) Telomere restriction fragment length measured in primary cultures from tumours in (**A**) (passage 3, P3) and the previous experiment (passage 2, P2) by Southern blot. Telomeres of treated UXF1138L xenografts were ∼1 kb shorter than control tissues (TRF P2, lane 2: ∼5.7 kb compared to lane 3: ∼4.7 kb; TRF P3, lane 4: ∼4.6 kb *vs* lane 5: ∼3.4 kb). Lane 1=molecular weight standard supplied with the Roche Telo-TAGGG kit. (**D**) Loss of nuclear hTERT expression and occurrence of atypical mitotic figures after RHPS4 treatment. Control tissues were probed with mouse IgG (isotype negative control, (**a**), and monoclonal hTERT antibodies (**b**). RHPS4-treated tissue was stained for hTERT protein expression (**c**), sections were counterstained with haematoxylin. RHPS4 treatment leads to the loss of nuclear hTERT expression (**c**) and increase in mitotic abnormalities, for example ring chromosomes (enlargement and black arrows) and anaphase bridges.

RHPS4 was administered orally twice a week for the course of the experiment at half of its maximal tolerated dose (5 mg kg^−1^ day^−1^). RHPS4 at 5 mg kg^−1^ day^−1^ was well tolerated in all *in vivo* studies and did not cause any noticeable side effects, such as body weight loss (Table 1). Efficacy of RHPS4 in subcutaneously growing UXF1138L xenografts was determined in terms of ‘bulk’ tumour growth inhibition relative to vehicle-treated controls (Figure 3A) as well as by measuring clonogenicity. As shown in Figure 1, control-treated and RHPS4-treated UXF1138L tumours were transplanted into new animals upon termination of a therapy experiment and treatment was essentially continued in another host. Engraftments of treated tumour tissues were performed for four consecutive passages. The result for single-agent RHPS4 in passages 1–4 are summarised in Table 1. Because UXF1138 xenografts are fast growing (average tumour doubling time=5 days), we had to employ serial transplantation of RHPS4-treated tissues to evaluate pharmacodynamic end points that would likely require ‘chronic’ drug exposure such as successive telomere erosion and inhibition of G_0_-arrested tumour stem cell fractions. The results of the single-agent study in passage 3 are shown in Figure 3A. Although, RHPS4 did not show oral single-agent activity in any of the four passages, we did observe marked reduction in clonogenicity of RHPS4-treated tumour tissue in the soft agar tumour stem cell assay; inhibition of stem cell growth increased with successive passages (Table 1). In the experiment depicted in Figure 3, we found tumour growth inhibition to a maximal extent of 33% (optimal T/C at day 28 was 67%, *P*=0.02) compared with control, but a significant inhibition of colony forming units in the same tumour tissues of 54%±6.6 (*P*<0.0028) (Figure 3A, insert).

### Single-agent RHPS4 modulates telomeres and telomerase *in vivo*

DNA generated from primary cultures of RHPS4-treated tumour tissues that were harvested at termination of each experiment (see Figure 1) was analysed for telomere length (Table 1). As shown for passages 2 and 3 a clear difference between TRF length of control and treatment groups was observed (Figure 3C). The mean telomere length in RHPS4-treated xenograft tissue was approximately 1 kb lower than in control tissues (Table 1). Overall, TRF length appeared to shorten at a rate of 1 kb per passage (∼28 days). It has to be noted that accurate measurement of telomere length of primary cultures from xenografts is problematic, because the cultures contain a mix of human cancer cells and murine cells. As seen in Figure 3C, an additional strong very high TRF signal (>21 kb) representing mouse telomeres was detected. Compared to the TRF length of pure human UXF 1138L cells growing in tissue culture (2.7 kb), the primary cells derived from *in vivo* grown UXF1138L tumours had longer telomeres that varied in control cultures from passage to passage (Table 1 and Figure 3C). This is likely due to contamination with mouse cells.

**Figure 5:**
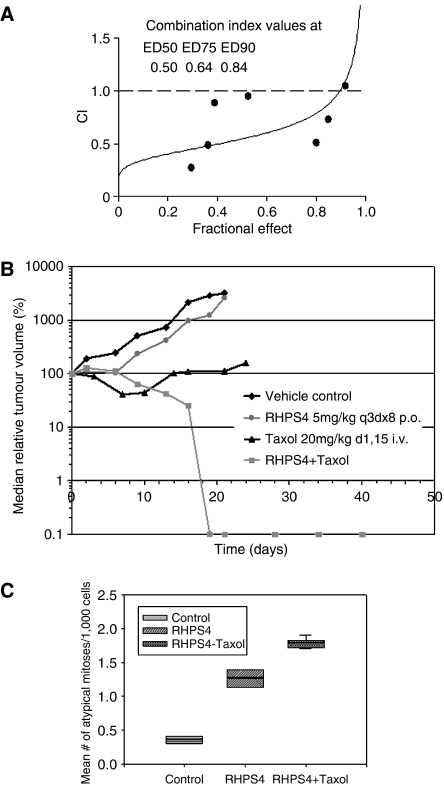
(**A**) The combination of RHPS4 and Taxol in UXF 1138L cells *in vitro* is synergistic. Shown are the CI against fractional effect (on growth) for the *in vitro* combination of RHPS4 and Taxol at a fixed ratio of their individual IC_50_ values. CI values are given for the doses effecting 50, 75 and 90% growth inhibition compared with control (ED_50_, ED_75_ and ED_90_, respectively); CI values below 1 indicate synergistic drug effects (Chou and Talalay, 1984). (**B**) Tumour growth inhibition of UXF1138L xenografts by Taxol given at 20 mg kg^−1^ i.v. on days 1 and 15. Shown is the median relative tumour volume in %. Control and RHPS4 groups had to be sacrificed on day 21, whereas the combination showed complete remissions and was terminated after 40 days. Minor remissions were seen on days 7–10 (*n*=6 mice). The combination of RHPS4 (5 mg kg^−1^ p.o. twice weekly) and Taxol (single dose 20 mg kg^−1^ i.v. on day 1) was highly effective and led to complete, durable remissions of UXF1138L xenografts. RHPS4 alone produced only marginal growth inhibition (*n*=5 mice). (**C**) Box plots for atypical mitosis in UXF1138L tumours. Residual tissues masses from RHPS4/Taxol treated tumours show pronounced induction of atypical mitoses compared to vehicle control. The number of mitotic abnormalities is further increased in the combination group from that seen with single-agent RHPS4. Control=0.35±0.07, RHPS4 alone=1.25±0.19, and RHPS4+Taxol=1.8±0.08. The line within the box marks the median, whiskers indicate the 10th and 90th percentiles of the box plots.

Control and treated UXF1138L xenograft tissues were also analysed for hTERT protein expression (Figure 3Da–c). Control tumour tissue (Figure 3Db) readily expressed nuclear hTERT with an accumulation of the enzyme in the nucleoli. In RHPS4-treated UXF1138L xenograft tissue, loss of strong nuclear hTERT expression was observed, but weak nuclear and cytoplasmic hTERT staining remained (Figure 3Dc). Isotype control antibody-stained sections were completely negative (Figure 3Da), confirming that the weak hTERT protein expression is specific. Reduced-hTERT expression was accompanied by the prominent occurrence of atypical mitotic figures such as ring chromosomes (Figure 3Dc, enlargement) and anaphase bridges, indicative of telomere dysfunction and chromosomal damage. Atypical mitotic figures were quantified in Figure 5C. RHPS4 mono therapy (5 mg kg^−1^ day^−1^ p.o.) evoked a significant induction of mitotic abnormalities compared to vehicle-treated control (*P*=0.0011).

### Evidence of telomere uncapping by RHPS4 *in vitro*

**Figure 4:**
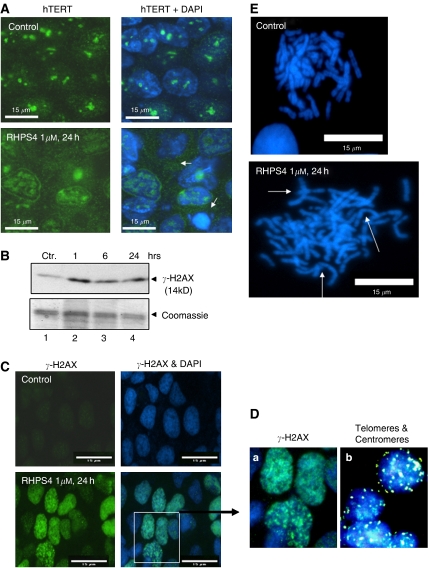
(**A**) Expression of hTERT in UXF1138L cells. Expression of hTERT in UXF1138L cells treated with PBS (control) or 1 *μ*M RHPS4 for 24 h. In RHPS4-treated cells, nuclear hTERT signal is attenuated. The white arrows in the lower panel to the right indicate distribution of hTERT in the cytoplasm. Cells were dual labelled against hTERT (green) and for DNA (blue). Bars=15 *μ*m. (**B**) Western blot of nuclear extracts from UXF1138L cells treated for 1, 6 and 24 h with 1 *μ*M of RHPS4. Membranes were developed with anti-*γ*-H2AX antibodies (upper panel), and/or gels directly stained with Coomassie blue (lower panel, equal loading control). (**C**) *γ*-H2AX expression in nuclei of UXF1138L cultured in the absence (top) and presence of RHPS4 (bottom). (**D**) Enlargement of *γ*-H2AX positive, DAPI-stained UXF1138L cells from (**D**) (indicated by white box) in (**a**), and UXF1138L interphase nuclei probed with human telomere (pink) and centromere (green) paints by fluorescence *in situ* hybridisation in (**b**). (**E**) Metaphase spreads from treated (24 h) and control UXF1138L cells. RHPS4 exposure for 24 h (1 *μ*M) results in ring and dicentric chromosomes (white arrows) that are responsible for the formation of anaphase bridges.

To confirm and clarify the data presented in Figure 3D, we followed hTERT protein expression after treatment with 1 *μ*M RHPS4 in UXF1138L cells *in vitro.* Control cells exhibited strong expression of hTERT in the nucleoplasm particularly in the nucleoli (Figure 4A); nuclear hTERT expression was attenuated, whereas cytoplasmic protein was more detectable in cells treated with RHPS4 for 24 h (Figure 4A, white arrows). This suggests that RHPS4 binding to the telomere can displace hTERT from the nucleus leading to its translocation into the cytoplasm. Concomitantly, we observed the phosphorylation of histone variant H2AX, *γ*-H2AX (Figure 4B and C), which indicates putative telomere-initiated DNA-damage signalling. *γ*-H2AX expression was seen as early as 1 h after exposure of UXF1138L cells to 1 *μ*M RHPS4 by Western blot and at similar levels at 6 and 24 h (Figure 4B), suggesting the maximal signal was reached at 1 h already. We confirmed the 24 h time point by immunoflourescence staining of *γ*-H2AX foci (Figure 4C). The majority of RHPS4-treated UXF1138L cells showed strong *γ*-H2AX foci formation that were extended throughout the nucleus (Figure 4Da), a smaller fraction of nuclei showed a distinct punctuate *γ*-H2AX pattern (Figure 4C and Da). To investigate whether *γ*-H2AX foci might localise to telomeres, we performed fluorescence *in situ* hybridisations with telomere and centromere probes on interphase nuclei of UXF1138L cells (Figure 4Db). Because of the very short telomere length in UXF1138L cells, telomere signal (pink, Figure 4Db) was very weak, but a clear punctuate pattern was observed that did not match to the more diffuse and extensive *γ*-H2AX foci. Moreover, the number of centromere (green) and telomere signals (pink, Figure 4Db) was consistent with the number of chromosomes, whereas *γ*-H2AX foci exceeded the number of telomeres.

During the microscopic evaluation of *γ*-H2AX foci, it became apparent that RHPS4-treated UXF1138L had an increased occurrence of anaphase bridges (data not shown). To test whether anaphase bridges are a result of chromosome fusions, we generated metaphase spreads from control cells and cells treated with 1 *μ*M RHPS4 for 24 h (Figure 4E). DAPI staining revealed that RHPS4 has a marked effect on chromosome morphology; an increase in end-to-end joining, as evident in ring and dicentric chromosomes were observed (Figure 4E, white arrows). The very rapid occurrence of RHPS4 effects depicted in Figure 4 strongly supports the hypothesis that RHPS4 can cause telomere-capping alterations in tumour cells with short telomeres such as UXF1138L.

### RHPS4 and Taxol act synergistically

Under the influence of a mitotic spindle poison (Taxol stabilises microtubles), mitotic cells fail to enter anaphase. This mechanism together with the telomeric DNA-damage response induced by RHPS4, which leads to anaphase bridging (see Figure 4E), suggested us that the two agents might synergise. First, we performed *in vitro* cytotoxicity assays in UXF1138L cells with the single agents and the combinations thereof at their fixed IC_50_ values, and processed the results using Calcusyn software. Taxol combined with RHPS4 showed combination indices (CI) below 1 at all levels (%) of effect, ED50 (ED, effective dose), ED75 and ED90, indicating synergism of the two drugs (Figure 5A). Second, we combined RHPS4 with Taxol *in vivo* and evaluated UXF1138L tumour growth inhibition in nude mice. The study was performed with UXF1138L tumours in passage 4 (Figure 1B) of continuous treatment with RHPS4 and not previously untreated UXF1138L tumours because we wished to continue to study single-agent activity with successive passages and exploit the concept of RHPS4 as a chemosensitising agent; RHPS4 was given as detailed above (see Figure 3). The combination of RHPS4 and Taxol together showed markedly enhanced efficacy over that of either single agent alone (Figure 5B, Table 1). Taxol alone produced significant growth inhibition (optimal T/C (day 21)=8%, *P*<0.04) with transient remissions seen on days 7–10 when the drug was given i.v. on days 1 and 15. RHPS4 single-agent activity was slightly more pronounced than in passage 3 (Figure 3) with an optimal T/C of 62% (Figure 5B). For the *in vivo* combination, we administered RHPS4 at 5 mg kg^−1^ p.o. twice weekly till the experiment was terminated (day 40, Figure 5B) and injected Taxol i.v. (20 mg kg^−1^=MTD) together with the first dose of RHPS4. A second dose of Taxol on day 15 was not given, because the tumours had regressed (T/C day 15=1%, Figure 5B). Complete remissions were observed as of day 19 (T/C=0%, *P*<0.0017). The combination regimen and both of the single agents were well tolerated and appeared to lack noticeable, side effects. No body weight loss or drug-related deaths were observed (Table 1). We have used groups of 5–6 animals with two subcutaneously growing xenografts each (*n*=10–12 tumours). Individual animals in the combination group had residual tumour masses (smaller than the tumour size at day 0 of the experiment, Table 1), which were excised and analysed for mitotic abnormalities. UXF1138L vehicle control tumours and xenografts treated with RHPS4 alone were also examined. As seen before for the single-agent treatment, anaphase bridging and atypical mitoses occurred (Figures 3D and 5C, *P*<0.001). They were even more pronounced in the combination group (Figure 5C, *P*<0.0003). Together, our *in vitro* and *in vivo* data suggest that Taxol and RHPS4 could be useful clinical combination partners.

## DISCUSSION

Here we provide evidence for phenotypic effects consistent with telomere uncapping induced by the G-quadruplex ligand RHPS4 as the mechanism for *in vitro* and *in vivo* anti-tumour activity. Our data showing the loss of the telomerase catalytic subunit hTERT from the nucleus (Figure 4A) and the rapid induction of putative telomere-initiated DNA-damage signalling as indicated by *γ*H2AX phosphorylation support the hypothesis that RHPS4 targets both telomeres and telomerase. The loss of telomere-associated proteins that have capping function such as hTERT and the stabilisation of G-quadruplexes at the telomeric G-strand overhang upon ligand binding appears to be more detrimental to cancer cells than normal cells that express telomerase (Figure 2). Normal cell types expressing telomerase are those with self-renewal capacity; they include germ cells, embryonic stem cells and adult stem cells (Holt *et al*, 1996; Burger, 1999). To examine the specificity of RHPS4 for cancer cells, we have cultured the human embryonic kidney cell line, HEK293T, and human cord blood in clonogenic assays, which are known to grow stem cells (Hamburger and Salmon, 1977; Fiebig *et al*, 2004). Interestingly, as shown in Figure 2C, colony forming units of cord blood and HEK293T cells were over a log-fold less sensitive to RHPS4 treatment than colonies forming from tumour cells (Figure 2A and B). Cell kill of normal stem cells was only seen at high drug concentrations (∼10 *μ*M) suggesting that RHPS4 might have a relatively wide therapeutic window. Moreover, at RHPS4 concentrations that markedly inhibited tumour colony forming units (0.1–1 *μ*M), cord blood and HEK293T-derived colony growth was induced. When clonogenic growth of human tumour cells was compared to bulk cell growth, pronounced differences were seen (Figure 2A and B). Whole-cell populations were more resistant to RHPS4. These observations strongly suggest that human tumour stem cells can be differentially targeted by G-quadruplex stabilising ligands and are in agreement with recent findings that hTERT is a ‘stemness’ gene: hTERT overexpression has been found to promote stem cell mobilisation, whereas short telomeres have been reported to cause stem cell failure (Hao *et al*, 2005; Sarin *et al*, 2005). In cancer, stem cells are best understood in haematological malignancies. Whereas telomere length maintenance in primitive human haematopoietic cells is dissociated from telomerase activity, telomerase-dependent telomere shortening appears to be involved in the chromosomal instability and transformation of haematopoietic stem cells into leukaemia stem cells (Wang *et al*, 2005; Ju and Rudolph, 2006). Despite the inherent presence of telomerase in normal stem cells, cancer stem cells arising from the latter require markedly higher telomerase levels that are more efficient at telomere maintenance (Armanios and Greider, 2005). Thus, cancer stem cells might be more susceptible to loss of functional telomerase by telomere uncapping.

While telomerase expression and telomere maintenance are key to the limitless proliferative potential of stem cells, another key feature is their ability of self-protection. At a molecular level this is due to the expression of drug efflux pumps such as P-glycoprotein (Pgp) and breast cancer resistance protein (BCRP) (Goodell *et al*, 1996; Donnenberg and Donnenberg, 2005). The uterine carcinoma UXF1138L xenograft used in this study is overall resistant to standard chemotherapy including drugs that are substrates of Pgp and BCRP such as doxorubicin and mitoxantrone (Fiebig and Burger, 2001); only Taxol has single-agent activity and tumours inevitably re-grow after treatment (Figure 5B). This indicates that UXF1138L tumours contain cells that can escape cytotoxic therapy and re-populate the tumour consistent with the existence of cancer stem cells.

Although RHPS4 did not show significant single-agent activity in the regimen that we evaluated (optimal T/C 67%) according to criteria set by the US-NCI (optimal T/C 40%=efficacy, Alley *et al*, 2004), we did observe marked target effects, namely loss of hTERT expression in the nucleus, telomere shortening (∼1 kb over 28 days) and telomere uncapping as suggested by the occurrence of anaphase bridges or ring chromosomes (lack of significant single-agent activity might be due to poor oral bioavailability of the aridinium methosulfate salt, intravenous injections of RHPS4 might prove more effective) (Figure 3; Gisselsson *et al*, 2001). Most importantly, however, a significant reduction in clonogenicity of RHPS4-treated tumour tissue in the soft agar tumour stem cell assay was seen (Figure 3, inset). Thus, the striking efficacy and synergism between RHPS4 and Taxol, in the results shown in Figure 5, can be interpreted in light of two factors: firstly, mechanistic synergism between a mitotic spindle poison, under which mitotic cells fail to enter anaphase, and a telomere-damaging agent which induces anaphase bridging; and secondly, the combination of a debulking agent (Taxol), with an agent that can target critical ‘stemness factors’ in tumour stem cells (RHPS4).

Our observations and data interpretation are supported by several previous publications. First, mutant (dominant negative) hTERT-expressing cancer cells show reduced telomerase activity (inhibition), reduction in telomere length, chromosome fusions, slowing and eventual arrest of cell growth related to initial telomere length, and reduced tumourigenicity in immunodeficient nude mice (Hahn *et al*, 1999). Our own mutant-hTERT MCF-7 breast cancer cell line model also demonstrated markedly reduced clonogenicity and tumourigenicity (Cookson *et al*, 2005; data not shown). Second, GRN163L, a modified antisense oligonucleotide directed against the telomerase RNA component and the first telomerase inhibitor to enter clinical trials, produces *in vitro* telomerase inhibition, progressive telomere shortening, reduced clonogenicity and tumourigenicity of breast cancer cell lines, and suppression of tumour growth and lung metastases in animal models *in vivo* (Dikmen *et al*, 2005; Kelland, 2005; Hochreiter *et al*, 2006; Burger, 2007). Third, Gowan *et al* (2002) showed that after debulking a tumour with Taxol, re-growth was effectively prevented by subsequent treatment with the small molecule G-quadruplex binding ligand BRACO19.

However, not all cytotoxic drugs might be suitable as debulking agents and/or combination partners for RHPS4. We have previously evaluated a range of clinically approved and experimental anticancer agents in combination with RHPS4 in cancer cell lines *in vitro* using the combination index method by Chou and Talalay (1984). Although, the *in vitro* data demonstrated that RHPS4 can act synergistically with Taxol in the UXF1138L cell line (Figure 5), as well as in MCF-7 cells, DNA-cross linking and alkylating agents such as cisplatin and temozolomide were antagonistic, possibly because these agents preferentially react at G-rich DNA sequences. Synergism between RHPS4 and other drugs were observed if there was an overlap between the molecular mechanism of the combination partners (Cookson *et al*, 2005).

*In vitro* observations by Leonetti *et al* (2004) initially established a telomere uncapping effect of RHPS4, while Incles *et al* (2004) showed the same mechanism for another G-quadruplex ligand, BRACO19. Both agents caused DNA end-to-end joining as a result of 3–21 days treatment in prostate cancer and melanoma cell lines that had an average telomere length between 4–10 kb. The same phenomenon was seen by us in UXF1138L *in vitro* cultures, after just 24 h treatment with RHPS4 (Figure 4E). Exposure to 1 *μ*M RHPS4 for 24 h led to a marked increase in end-to-end joining, as evident in ring and dicentric chromosomes in metaphase spreads compared to vehicle controls. This earlier response to telomere dysfunction by UXF1138L cells compared to melanoma and prostate cancer cell lines might be due to their very short telomeres (2.7 Kb) and suggests that in proposed clinical trials with RHPS4, patient tumours should be tested for telomere length at the outset of therapy and considered as a putative predictive marker of response.

A DNA break within a chromosome is sensed by the DNA damage response machinery of the cell with the result that, following cell cycle arrest to allow time for the repair, the DNA break is fixed by end-to-end rejoining (Blasco, 2005). One of the earliest events at the site of DNA DSB is the phosphorylation of histone variant H2AX, *γ*-H2AX, on residue Ser139. The loss of telomere function by either gradual telomere shortening or uncapping (loss of binding proteins e.g. hTERT, TRF2) has been proposed to mimic DNA double-stranded breaks (d'Adda di Fagagna *et al*, 2003; Hao *et al*, 2004). We have tested whether a short term (24 h) treatment of UXF1138L cells (telomere length=2.7 Kb) with RHPS4 leads to *γ*-H2AX expression, indicating uncapping, and found indeed a rapid induction of *γ*-H2AX phosphorylation by RHPS4. The pattern of *γ*-H2AX foci formation suggests that RHPS4-induced DNA damage is not limited to telomeric DNA, but appears to extend beyond telomeric regions. This becomes evident from comparing the number of *γ*-H2AX foci after RHPS4 treatment to telomere signal in UXF1138L cells (Figure 4Da and b).

These phenomena are indicative of RHPS4 inducing G-quadruplex DNA formation in the telomeric sequence and causing displacement of the catalytic subunit from the telomere (Figure 4A). While we have focused on following the displacement of hTERT from the telomere (results herein; and Burger *et al*, 2005), Salvati *et al* (2007) have recently shown that RHPS4 also modulates other telomere binding proteins (TBP). This highlights the possibility that the detection of hTERT and/or TBP localisation together with the induction of *γ*-H2AX should be considered as surrogate markers for the response to telomere targeting agents, and that they might provide reliable and fast signals of target inhibition that could replace the need for post-therapeutic telomere length determination.

In summary our data indicate that the combination of RHPS4 and Taxol should be evaluated clinically for the treatment of tumours with short telomeres. The exploitation of pre-treatment telomere length should be integrated into the clinical trial designs along with post-treatment *γ*-H2AX phosphorylation or loss of telomere-binding proteins. We have presented intriguing evidence that RHPS4 can differentially inhibit the growth of clonogenic tumour cells, considered to be cancer stem cells. Effective tumour debulking by Taxol together with eradication of cancer stem cells by RHPS4 could explain the marked synergism of these two agents against UXF138LX xenografts *in vivo.* Further studies defining the efficacy of RHPS4 in targeting cancer stem cells, such as NOD/SCID mouse repopulation assays, are warranted.

## Figures and Tables

**Table 1:** Summary of *in vivo* efficacy and pharmacodynamics

**UXF1138L Xenograft**	**Opt. test/control (%) (day)**	**BWC (%)**	**Deaths (n/n)**	**HTCA growth (%)**		**TRF (kb)**
Passage #1						
Vehicle control	100 (0)	+9	0/5	100±5.2		6.0
RHPS4 5 mg kg^−1^	63 (16)	+26	0/5	83±2.3		5.1
					
Passage #2						
Vehicle control	100 (0)	+13	0/6	100±22		5.7
RHPS4 5 mg kg^−1^	100 (7)	+9	0/6	93±2.3		4.7
					
Passage #3						
Vehicle control	100 (0)	+29	0/8	100±35		4.6
RHPS4 5 mg kg^−1^	67.7 (28)	+21	0/8	54.5±6.6		3.4
					
Passage #4						
Vehicle control	100 (0)	+22	0/5	100±6.2		4.9
RHPS4 5 mg kg^−1^	62 (16)	+17	0/5	44±6.4		4.2
				**CR**	**PR**	**P**
Taxol 20 mg kg^−1^	8 (24)	+6	0/6	2/12	7/12	3/12
Taxol/RHPS4	0 (19)	+10	0/5	8/10	2/10	0/10

Opt. Test/Control (%) (day), optimal test/control median tumour volume in % and day it was observed; BWC, maximal median body weight change in %; n/n, number of drug-related death per number of mice per group; HTCA, growth in the human tumour colony assay, colony growth of control was set 100%; TRF, mean telomere restriction fragment length in kilo bases; CR, complete remission; PR, partial regression at any time during the experiment compared to initial tumour volume; P, progression.
